# Two new thematic series—spotlight on artificial intelligence and a specific platform for technologist

**DOI:** 10.1186/s41824-019-0067-1

**Published:** 2019-12-16

**Authors:** Francesco Giammarile, Riemer H. J. A. Slart, Pedro F. Costa

**Affiliations:** 1 Nuclear Medicine and Diagnostic Imaging Section, International Atomic Energy Agency, Vienna, Austria; 2 Medical Imaging Center, Department of Nuclear Medicine and Molecular Imaging, University of Groningen, University Medical Center Groningen, Groningen, The Netherlands; 3 TechMed Centre, Department of Biomedical Photonic Imaging, University of Twente, Enschede, The Netherlands; 4 Department of Nuclear Medicine, University Hospital Essen, University of Duisburg-Essen, Essen, Germany

Medical imaging techniques, such as computed tomography (CT), magnetic resonance imaging (MRI), positron emission tomography (PET) and single-photon emission computed tomography (SPECT) have acquired a central role in the clinical management of a wide variety of diseases. The unique advantage of imaging is the non-invasive characterisation and quantification of the presence of pathophysiological processes at different stages of diseases and the ability to monitor the disease. The advent of hybrid imaging PET/CT, SPECT/CT and PET/MRI brought the best of two worlds for the patient’s benefit, but it represents a continuous challenge in standardising and optimising methodologies for the health professionals responsible for imaging.

In this setting, we are pleased to announce the publication of two distinct thematic series, one dedicated to artificial intelligence (AI) and the second specifically dedicated to technologists.

Optimal use of hybrid and multimodality medical imaging data can maybe further improved by applying artificial intelligence, to speed-up and increase the diagnostic accuracy in various diseases and prediction of response on therapy. The term AI is applied when a device mimics cognitive functions, such as learning and problem solving. More generally, AI refers to a field of computer science dedicated to the creation of systems performing tasks that usually require human intelligence, branching off into different techniques. Machine learning, deep learning and artificial neural networks are those approaches that allow computers to learn from data and have emerged as promising in the fields of nuclear medicine and in radiology. The majority of the physicians are not experienced in AI applications, due to a gap in knowledge and lack of training facilities or some amongst us with disbelieve in or even fear AI. We however cannot ignore the development of AI systems in medical imaging, as is visible in the exploding number of publications and abstracts on medical imaging congresses, particularly in the field of radiology. Nuclear medicine is growing less rapidly in AI applications, probably due to a less amount of acquired data, but is rising as well, as the number acquisition procedures, in particular PET/CT, are growing.

So, should we see AI as an opportunity or as a threat in our profession? AI is believed to be a tremendous opportunity for its improvement. Similar to our natural intelligence, AI algorithms look at medical images to identify patterns after being trained using vast numbers of examinations and images. In this scenario, AI is not a threat to our imaging profession. With the irreversible increase in imaging data and the possibility to identify findings that humans can or cannot detect, AI may support you in the clinical practice. The awareness of inter- and intra-reader variability in medical imaging over the past decades proves the need for reproducible medical imaging results. Because of the rapid growth of this area, numerous published AI investigations lack standardised evaluation of both the scientific integrity and the clinical usefulness of AI investigation of imaging data, such as intensity, shape, texture and artefact corrections, that can be extracted from medical images and extracted by or integrated in machine learning approaches, providing valuable information for optimal diagnosis. Finally, AI applications may enhance the reproducibility of technical protocols, improving image quality and decreasing radiation dose, decreasing scanner time and optimising scanner utilisation, thereby reducing costs.

New results of running multi-center trials will provide the additional evidence needed for the potential real beginning of AI in the clinic, but the implementation of reliable software solutions from the imaging manufacturers is warranted.

We are at the beginning of the AI era, and it will surely impact nuclear medicine and radiology and even faster than other clinical fields (Table 1).

**Table 1:** List of topics in the artificial intelligence thematic series

1. Basics of AI applications in hybrid and multimodality imaging • Systematic review of AI application in hybrid and multimodality imaging • Clinical basics of AI in hybrid and multimodality imaging • Technical basics of AI in hybrid and multimodality imaging	
2. Clinical application of AI in in hybrid and multimodality imaging • AI in oncological applications o Diagnostics o Therapy • AI in cardiovascular applications • AI in neurological applications	

The second thematic series is both a pivotal work in the scientific engagement of clinical technologists and a frame that reflects the changes and challenges present on the field.

During the last decennia, ionising radiation procedures have undergone major transformations in regard to safety, equipment technologies, communication, scientific basis and clinical knowledge. The accentuated increase witnessed mainly in radiological procedures (computed tomography and magnetic resonance imaging) deeply influenced a generation of health professionals, amongst which is the nuclear medicine technologist (NMT).

Nuclear medicine procedures are on the interface of therapy and diagnosis in the clinical, preclinical and academic domains. Also here, technological development and radiation protection awareness has led to the revision of practices and procedure optimisation. Advances in radiochemistry knowledge have contributed significantly to the design of more favourable tracers, which have culminated at the theranostic pairs, widely used in diagnostic and therapy of a number of diseases.

In terms of scientific disciplines, nuclear medicine dwells at the junction between biochemistry, physics and medicine, with a strong pre-clinical component. Radioisotope production requires a deep knowledge of radiochemistry, and radiopharmaceutical synthesis is nowadays regulated as industrial pharmacology. Quality control and assurance, radiation safety and good clinical practice are nowadays an integral part of nuclear medicine and are fundamental for the functionality of modern nuclear medicine departments. Also placing the focus on patient comfort and involving patients in the clinical procedures has been a major change in the last decade. The special nature and development of nuclear medicine procedures has directly influenced NMT development.

In the academia domain, it seems that technologist involvement is unavoidable and this has been thoroughly acknowledged in scientific papers in many journals. However, it still seems quite difficult to have research activities initiated and managed by technologists. Specially in the field of nuclear medicine, the current publication possibilities are dominated by physicians, physicists and clinical scientists, and there is no dedicated editorial space for technologists within the EJNMMI journal family. Nevertheless, at the EANM annual congress, a considerable volume of high-quality scientific data is presented by NMT colleagues from all over Europe, which are, without a dedicated platform to publish their data, driven to publish their work outside the EJNMMI umbrella journals, hence motivating the development of a thematic series on technological requirements in hybrid imaging, being hosted by the European Journal of Hybrid Imaging (Table 2).

**Table 2:** List of topics in the technologist thematic series

1. Introduction a. Technologist competencies b. Radiation protection and exposure optimisation	
2. Methods a. PET/CT/MR b. SPECT/CT/MR c. Preclinical imaging	
3. Indications a. Optimising conventional NM (diagnostic and therapy) b. Myocardial perfusion imaging (MPI) c. Oncology d. Theranostics e. Emergent radiopharmaceuticals	

The aims of these thematic series are to provide a stage (1) to describe the background, value and application of AI in medical imaging, particularly in in the field of hybrid and multimodality imaging, and (2) sharing the current state of NMT competencies applied to hybrid imaging on the different valences of nuclear medicine and provide a stage of discussion on their future trends.

It is intended that this platform will be populated with AI and NMT publications, hopefully providing a trigger on the scientific potential that is acknowledged to, especially amongst the (young) technologist and the physician colleagues interested in AI and all health radiation clinical disciplines.

